# Desensitization of idiopathic pulmonary fibrosis fibroblasts to *Alternaria alternata* extract‐mediated necrotic cell death

**DOI:** 10.14814/phy2.13020

**Published:** 2016-11-15

**Authors:** Jintaek Im, Kyutae Kim, Ji Young Yhee, Scott M. O'Grady, Richard S. Nho

**Affiliations:** ^1^ Department of Medicine University of Minnesota Minneapolis Minnesota; ^2^ College of Biological Science University of Minnesota St. Paul Minnesota; ^3^ Department of Animal Science Integrative Biology and Physiology University of Minnesota St. Paul Minnesota

**Keywords:** *Alternaria alternata*, idiopathic pulmonary fibrosis, lung fibroblasts, necrosis, reactive oxygen species

## Abstract

*Alternaria alternata* is an allergenic fungus and known to cause an upper respiratory tract infection and asthma in humans with compromised immunity. Although *A. alternata's* effect on airway epithelial cells has previously been examined, the potential role of *A. alternata* on lung fibroblast viability is not understood. Since lung fibroblasts derived from patients with idiopathic pulmonary fibrosis (IPF) display a distinct phenotype that is resistant to stress and cell death inducing conditions, the investigation of the role of *Alternaria* on pathological IPF fibroblasts provides a better understanding of the fibrotic process induced by an allergenic fungus. Therefore, we examined cell viability of control and IPF fibroblasts (*n* = 8 each) in response to *A. alternata* extract. Control fibroblast cell death was increased while IPF fibroblasts were resistant when exposed to 50–100 *μ*g/mL of *A. alternata* extract. However, there was no significant difference in kinetics or magnitude of Ca^2+^ responses from control lung and IPF fibroblasts. In contrast, unlike control fibroblasts, intracellular reactive oxygen species (ROS) levels remained low when IPF cells were treated with *A. alternata* extracts as a function of time. Caspase 3/7 and TUNEL assay revealed that enhanced cell death caused by *A. alternata* extract was likely due to necrosis, and 7‐AAD assay and the use of sodium pyruvate for ATP generation further supported our findings that IPF fibroblasts become resistant to *A. alternata* extract‐induced necrotic cell death. Our results suggest that exposure to *A. alternata* potentially worsens the fibrotic process by promoting normal lung fibroblast cell death in patients with IPF.

## Introduction

Asthma is a chronic inflammatory airway disease. When airways become inflamed and swollen, the muscles around the airways can tighten, which makes it difficult for air to move in and out of the lungs, causing symptoms such as coughing, wheezing, shortness of breath and/or chest tightness (Zanini et al. 2010; Bonini and Usmani 2015). *Alternaria* species are considered some of the most important fungi that can cause allergic reactions in humans (Downs et al. 2001; Bush and Prochnau 2004). Among them, the exposure to the allergenic fungus *Alternaria alternata* has been linked to increased risk of asthma (O'Hollaren et al. 1991; Zureik et al. 2002), and recent evidence showed that exposure to *A. alternata* in US homes is associated with active asthma symptoms (Salo et al. 2006, 2006). Studies showed that *A. alternata* triggers an inflammatory process by releasing several cytokines, causing asthma (Kauffman et al. 2000; Leino et al. 2013). More than 30 potential toxic metabolites have currently been isolated from *A. alternata* (Scott 2001; Ostry 2008), and each *A. alternata* metabolite is known to have its unique pathogenic effects including cellular cytotoxicity, mutagenicity, and carcinogenicity (Schrader et al. 2006; Bensassi and Gallerne 2012). The role of *A. alternata* and its metabolites on the pathogenicity of asthma were partially demonstrated by inducing several proinflammatory cytokines in airway epithelial cells leading to initiation of T helper type 2 (Th2) differentiation, and the disruption of the bronchial epithelial barrier (Murai et al. 2012; Leino et al. 2013). However, these studies elucidated the pathological effects of *A. alternata* mainly on lung epithelial cells, and it is currently unclear how *Alternaria* affects lung fibroblast viability associated with lung fibrosis.

Idiopathic pulmonary fibrosis (IPF) is a deadly and progressive fibrotic lung disease with a 5 year mortality rate of 50–70% comparable to many cancers (King et al. 2011; Borensztajn et al. 2013). It is characterized by the accumulation of fibroblasts and collagen within the alveolar wall resulting in obliteration of the gas‐exchange surface (King et al. 2011; Borensztajn et al. 2013). Although IPF pathogenesis is not fully understood, IPF is thought to be caused by chronic lung injury followed by an aberrant repair process. Studies have demonstrated that fibroblasts derived from patients with IPF maintain their apoptosis‐resistant phenotype in response to cell death inducing stimuli such as Fas ligand (FasL) and collagen rich matrix, etc. (Nho et al. 2011, 2013; Im et al. 2015, 2016). Interestingly, a recent study documented that *A. alternata* infection is also associated with lung fibrosis (Doherty et al. 2012), suggesting that the exposure to *A. alternata* may promote IPF. Since fibroblasts derived from lung specimens of IPF patients display a distinctive phenotype that is resistant to stress, environmental insults, and IPF has variable histologic features of inflammation and fibroproliferation (King et al. 2011; Borensztajn et al. 2013), the investigation of the role of *A*. *alternata* on normal and pathologically altered fibroblasts can be informative in understanding lung fibrosis.

Therefore, to test this concept, we first measured control and IPF fibroblast cell viability in response to *A. alternata* extract, and found that IPF fibroblasts are more resistant to *A. alternata* extract‐induced cell death as a function of time. However, there was no significant difference in kinetics or magnitude of Ca^2+^ responses from normal lung and IPF fibroblasts following *A. alternata* extract exposure. In contrast, IPF fibroblasts generated lower intracellular reactive oxygen species (ROS) levels in response to *A. alternata* extract, and the treatment of ROS scavenging chemicals predominantly affected control fibroblast viability, suggesting that lower ROS production is linked to enhanced IPF fibroblast viability in response to *A. alternata* extract. Our additional 7‐AAD assay, the use of sodium pyruvate for ATP generation, and caspase 3/7 inhibitor assay consistently suggest that cell death in lung fibroblasts caused by *A. alternata* extract occurs by necrosis, and IPF fibroblasts become resistant to this condition. Our results suggest that chronic exposure to *A. alternata* may increase the destruction of lung parenchyma by the induction of necrotic cell death of normal lung fibroblasts, and the presence of persistent fibrotic lung fibroblasts may exacerbate IPF.

## Materials and Methods

### Human subjects

Primary human lung fibroblast cells were generated from lung tissues removed at the time of transplantation or death from non‐IPF and IPF patients. The tissue samples were stripped of all identifiers and designated as waste (exemption 4). Exemption 4 includes research involving the collection or study of existing data, documents, records, pathological specimens, or diagnostic specimens, if these sources are publicly available or if the information is recorded by the investigator in such a manner that subjects cannot be identified, directly or through identifiers linked to the subjects. All patients underwent procedures for diagnostic or therapeutic procedures. Written informed consent was obtained on all patients prior to the procedure being performed. Use of human lung tissues was approved by the Institutional Review Board (IRB) at the University of Minnesota. The diagnosis of IPF was supported by history, physical examination, pulmonary function tests, and typical high‐resolution chest computed tomographic findings of IPF. In all cases, the diagnosis of IPF was confirmed by microscopic analysis of lung tissue that demonstrated the characteristic morphological findings of usual interstitial pneumonia (Xia et al. 2008).

### Preparation of primary lung fibroblasts

Eight pairs of control and IPF lung fibroblasts were prepared using individual tissues from non‐IPF and IPF patients by explant culture. Briefly, the removed lung tissues were chopped to 5 mm size and cultured in Dulbecco's modified Eagle's medium (DMEM; Sigma–Aldrich, St. Louis, MO) supplemented with 20% fetal calf serum (FCS; HyClone, Logan, UT) and 2% antibiotics for 4–5 weeks at 37°C in a 5% CO_2_ humidified incubator. Since the phenotype of lung fibroblasts could be altered at higher passage, we used cells having passages 3 through 8 in our experiments.

### Reagents and chemicals

*A. alternata* extracts were purchased from Greer labs (catalog no. XPM1C3A25, Lenoir, NC) and dissolved in serum‐free (SF) DMEM. Treatment of the same fibroblasts with a different lot number of *A. alternata* extracts resulted in different cell viabilities. Therefore, the optimal doses of *A. alternata* extracts from each lot were determined based on cell viability (i.e., when control and IPF fibroblasts were treated with 100 *μ*g/mL of lot no. 169626 and 400 *μ*g/mL of lot no. 276920, a similar effect on their cell viability was observed). The soluble form of recombinant FasL (catalog No. ALX‐522‐020‐C005) was purchased from Enzo Life Sciences (Farmingdale, NY). N‐acetyl‐L‐cysteine (NAC), Thapsigargin, Resveratrol, *tert*‐Butyl hydroperoxide (tBHP), and sodium pyruvate were purchased from Sigma–Aldrich. BAPTA‐AM and Caspase 3/7 inhibitor I was obtained from Biovision Inc. (Milpitas, CA) and EMD Millipore (Billerica, MA), respectively. Hank's balanced salt solution (HBSS) and acetoxymethyl ester form of Fura‐2‐AM were purchased from ThermoFisher Scientific (Pittsburgh, PA) and Invitrogen/Life Technologies (Carlsbad, CA), respectively.

### Cell viability assay

Randomly selected eight IPF and eight control fibroblasts (1 × 10^4^ cells/each well of a 96 well plate) were cultured in DMEM supplemented with 10% FCS and 1% antibiotics for 24 h for cell's initial attachment and growth. The next day, culture media were exchanged with SF DMEM and cells were cultured for an additional 24 h. Cells were then treated with 0–400 *μ*g/mL of *A. alternata* extract (0–100 *μ*g/mL for lot no. 169626 and 0–400 *μ*g/mL for lot no. 276920) for 1 h and then incubated with 20 *μ*L of Cell Titer Blue reagent (Promega, Madison, WI) for 1–7 h. Cell viability was measured at 560 nm (Excitation)/590 nm (Emission) of fluorescence using a 96‐well plate reader (BioTek, Winooski, VT).

### TUNEL assay

Terminal deoxynucleotidyl transferase dUTP nick‐end labeling (TUNEL) assay was conducted using HT TiterTACS^™^ assay kit (Trevigen, Gaithersburg, MD) according to manufacturer's instructions. Briefly, 1.5 × 10^4^ IPF and control fibroblasts (*n* = 3 each) were cultured on a 96 well plate under the aforementioned conditions and then exposed to 400 *μ*g/mL of *A. alternata* extract (lot no. 276920) for 0.5 h or FasL at 500 ng/mL for 24 h. After the incubation, cells were washed with PBS and fixed with 3.7% formaldehyde solution for 7 min at room temperature. After washing with PBS, cells were sequentially incubated in methanol for 20 min and Cytonin solution for 15 min. In order to quench endogenous peroxidase activity, cells were treated with 3% hydrogen peroxide solution for 5 min and washed with distilled water. After the incubation with 1 ×  TdT labeling buffer for 5 min, cells were incubated in labeling reaction mix for 1 h at 37°C, and the reaction was stopped by incubation in 1 ×  TdT stop buffer for 5 min. After washing with PBS, cells were sequentially incubated with Strep‐HRP for 10 min and TACS‐Sapphire solution for 30 min at room temperature. TUNEL‐positive cells were measured at 450 nm of absorbance using a 96‐well plate reader (BioTek) after adding 0.2 N HCl. As a positive control, cells were treated with TACS‐nuclease provided in the assay kit for 1 h at 37°C before hydrogen peroxide treatment.

### Caspase 3/7 activity assay

Caspase 3/7 activity was measured using Apo‐ONE Homogeneous caspase 3/7 assay kit (Promega). 1.0 × 10^4^ control and IPF fibroblasts (*n* = 8 each) were cultured on a 96 well plate in the presence of serum for 24 h followed by additional incubation with SF DMEM for 24 h. Cells were then treated with 100 *μ*g/mL of *A. alternata* extract (lot no. 169626) for 8 h at 37°C and then incubated with caspase enzyme substrate diluted in caspase 3/7 buffer for an additional 1 h at room temperature with continuous mixing. Caspase 3/7 activity was measured at 499 nm (Excitation)/521 nm (Emission) using a 96‐well plate reader (BioTek). For the positive control, cells were treated with 500 ng/mL of FasL and caspase 3/7 activity was measured at 24 h after FasL treatment.

### Confocal microscopic analysis for 7‐AAD staining

An apoptosis/Necrosis detection kit (Abcam, Cambridge, MA) was used to measure *A. alternata* extract*‐*induced cell death in control and IPF fibroblasts. 5 × 10^4^ control and IPF fibroblasts (*n* = 8 each) grown on a cover slip were serum starved for 24 h and then treated with 100 *μ*g/mL of *A. alternata* extract (lot no. 169626) for 0–40 min at 37°C. After washing with SF DMEM, cells were incubated with 7‐AAD solution for 30 min at room temperature and fixed with 2% paraformaldehyde solution for an additional 20 min. The coverslips were placed on slides containing Prolong Gold antifade reagent (Invitrogen/Life Technologies) to stain nucleus with DAPI. Fluorescence images for 7‐AAD and DAPI were obtained using an Olympus FluoView FV1000 BX2 Upright confocal microscope (Olympus, Tokyo, Japan) with 10× magnification at 546 nm (Excitation)/647 nm (Emission). Quantification of necrotic cells was performed using an image analysis software (Image‐Pro Plus 4.1, Media Cybernetics Inc., Rockville, MD), and values were presented as the percentage of 7‐AAD‐positive cell numbers against a total DAPI‐positive cell numbers on same image.

### Reactive oxygen species assay

Control and IPF fibroblasts (*n* = 8 each; 1 × 10^4^ cells/well of 96 well plate) were grown in serum containing medium for 24 h. Cells were then cultured for 24 h in SF DMEM and stained with 25 *μ*mol/L of 2′,7′‐Dichlorofluorescin (DCFDA, Sigma–Aldrich) for 50 min at 37°C. After washing with SF DMEM three times, cells were treated with 100 *μ*g/mL of *A. alternata* extract (lot no. 169626) and ROS levels were measured at 485 nm (Excitation)/535 nm (Emission) using a 96‐well plate reader (BioTek) every 5 min for 30 min after the treatment.

### Intracellular ATP assay

ATP levels were measured using a Luminescent ATP detection assay kit (Abcam) according to manufacturer's instructions. Briefly, control and IPF fibroblasts (*n* = 3 each, 1.5 × 10^4^ cells/well) cultured on a 96 well plate in SF DMEM for 24 h were treated with 400 *μ*g/mL of *A. alternata* extract (lot no. 276920) for 0.5 h at 37°C in the presence or absence of sodium pyruvate pretreatment at 0–20 mmol/L for 3 h. After treatment, cells were sequentially incubated with detergent for 5 min and substrate solution for 5 min on an orbital shaker. The plate was kept in a dark place for 10 min and luminescence was measured from individual wells for 500 msec using a 96‐well plate reader (BioTek).

### Intracellular calcium assay

The effect of *A. alternata* extract on intracellular Ca^2+^ concentration ([Ca^2+^]_i_) was performed as previously described (O'Grady et al. 2013). Briefly, control and IPF fibroblasts (*n* = 3 and 4 each) cultured in serum containing DMEM were plated on chamber slides in SF DMEM overnight. Before loading the cells with Fura‐2‐AM, the media was replaced with HBSS solution containing 10 mM HEPES buffer. After incubation with 5 *μ*mol/L Fura‐2‐AM for 1 h, cells were washed three times with HBSS and stimulated with 100 *μ*g/mL of *A. alternata* extract (lot no. 169626). Fluorescence was measured using a Diaphot inverted fluorescence microscope (Nikon, Tokyo, Japan) with 20× magnification at 340 and 380 nm (Excitation)/510 nm (Emission). Image acquisition and data analysis were performed using Image‐1 MetaMorph software (Universal Imaging Corporation, West Chester, PA). [Ca^2+^]_i_ was determined from the fluorescence ratio (*F*_*340*_ */F*_*380*_) following calibration with the Fura‐2‐AM calcium imaging calibration kit (ThermoFisher Scientific).

### Statistical analysis

**Figure 1:**
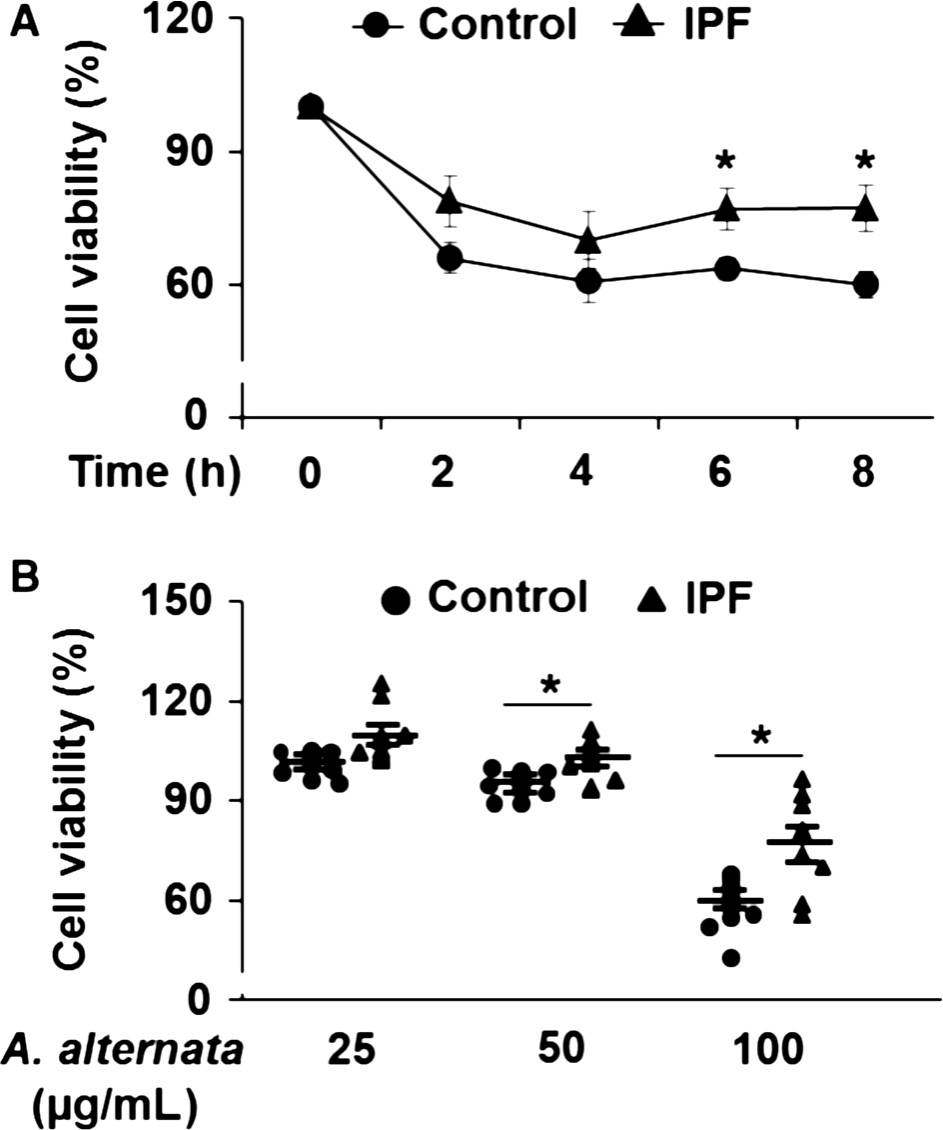
Enhanced resistance of idiopathic pulmonary fibrosis (IPF) fibroblasts to the *A. alternata* extract‐induced cell death. Randomly selected control and IPF fibroblasts (*n* = 8, each) were stimulated with 100 *μ*g/mL of *A. alternata* extract (lot no. 169626) as a function of time (A) or with various doses of *A. alternata* extract (lot no. 169626) for 8 h (B). Values given in A are the average ± SEM as the percentage against their respective nontreatment group (0 h), set at 100%. On the scatterplot in B, each circle and triangle point represents the percentage of viability of each control and IPF cell in response to *A. alternata* extract against their respective nontreatment groups (0 *μ*g/mL), set at 100%. (*) indicates statistical significance between control and IPF fibroblasts at *P *<* *0.05.

All data are presented as the mean ± SEM., and group comparisons between IPF and control, or nontreatment and treatment were carried out using Student's t‐test. The difference of % viability in control and IPF fibroblasts in the presence or absence of various chemicals was determined by Student's t‐test. In Figure 1B, a Welch correction was applied to determine the difference of viability in control and IPF fibroblasts at various doses of *A. alternata* extract. The significance level was presented as *P < 0.05, **P < 0.01 and ***P < 0.001.

## Results

### IPF fibroblasts showed enhanced resistance to the *A. alternata* extract‐induced cell death

Our previous studies demonstrated that lung fibroblasts from IPF patients maintain aberrantly high resistance to various cell death‐inducing conditions such as collagen matrix and FasL (Nho et al. 2011, 2013; Im et al. 2015, 2016). *A. alternata* and its metabolites have cytotoxic effects on a broad range of cell types (Wang et al. 1996; Cheng et al. 2011; Zhang et al. 2011; Bensassi and Gallerne 2012), and the presence of persistent fibrotic fibroblasts in response to cell death‐inducing environments is implicated with the progression of lung fibrosis. Therefore, we sought to investigate whether IPF fibroblasts are resistant to the cytotoxic effects of *A. alternata*, which may lead to the development of lung fibrosis. To test this possibility, cell viability was measured in randomly selected control and IPF lung fibroblasts (*n* = 8 each) as a function of time in the presence of *A. alternata* extract. Cell viability decreased by ~25% at 2 h and slightly decreased thereafter up to 8 h after 100 *μ*g/mL of *A. alternata* extract treatment in both control and IPF fibroblasts (Fig. 1A). Interestingly, IPF fibroblasts maintained enhanced cell viability in response to *A. alternata* extract compared to control fibroblasts at 6 and 8 h (Fig. 1A). Furthermore, when control and IPF fibroblasts were treated with various doses of *A. alternata* extract for 8 h, enhanced viability was also observed in IPF fibroblasts in response to 50 and 100 *μ*g/mL of *A. alternata* extract compared to control fibroblasts (Fig. 1B). These findings showed that *A. alternata* extract predominantly increases control lung fibroblast cell death.

### IPF fibroblasts are resistant to *A. alternata* extract‐induced necrotic cell death

**Figure 2:**
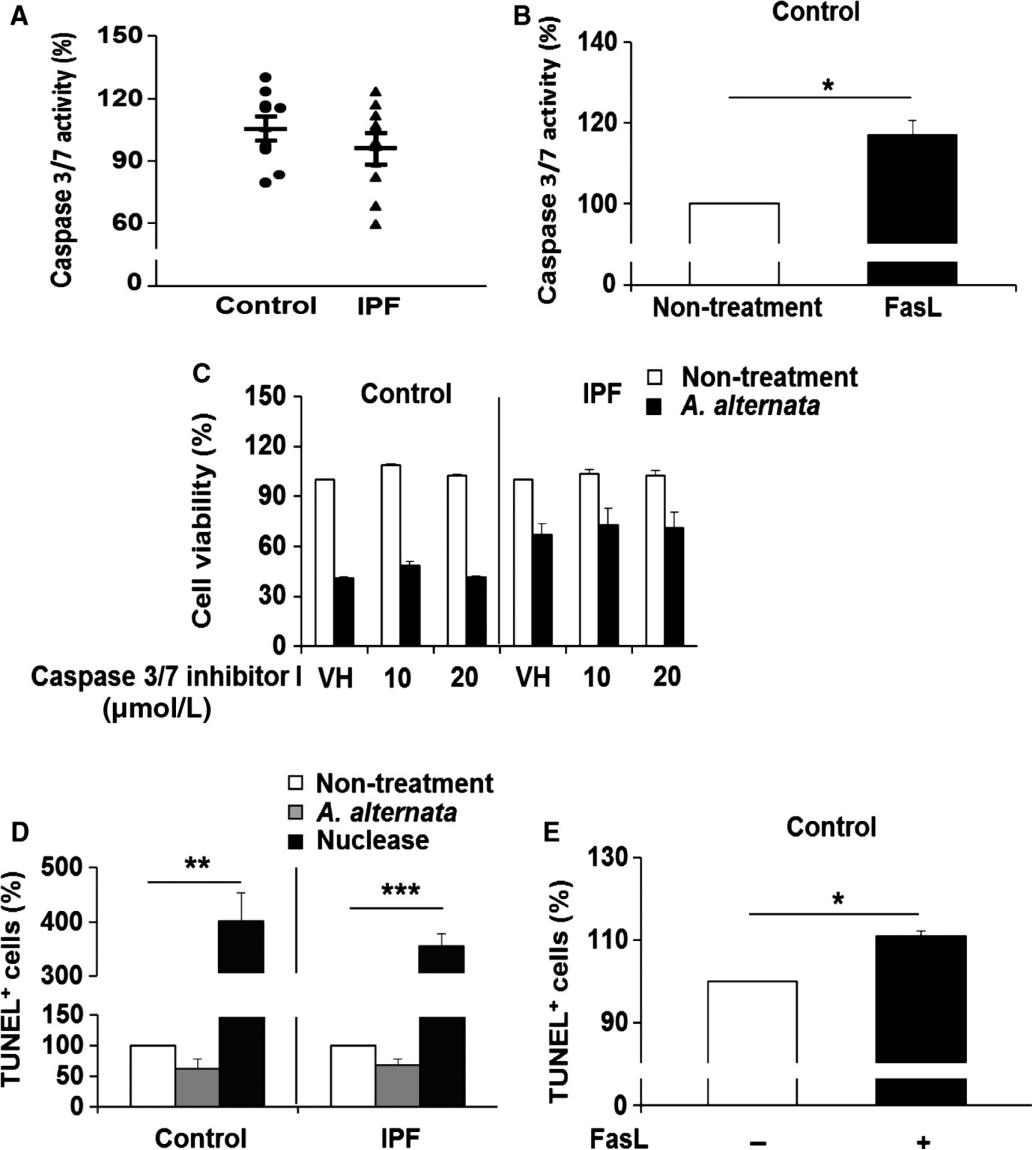
*A. alternata* extract causes nonapoptotic cell death in lung fibroblasts. (A) Control and idiopathic pulmonary fibrosis (IPF) fibroblasts (*n* = 8, each) were treated with 100 *μ*g/mL of *A. alternata* extract (lot no. 169626) for 8 h, and caspase‐3/7 activity was measured as described in the Materials and Methods. On the scatterplot, each circle and triangle point represents the percentage of caspase 3/7 activity of each control and IPF fibroblast in the presence of *A. alternata* extract against their individual nontreatment group, set at 100%. (B) Control fibroblasts (*n* = 3) were treated with 500 ng/mL of FasL for 24 h, and caspase 3/7 activity was measured. (*) indicates a statistical significance at *P *<* *0.05. (C) Control and IPF fibroblasts (*n* = 3, each; 1.0 × 10^4^ cells/well) preincubated with caspase 3/7 inhibitor I for 1 h were treated with 100 *μ*g/mL of *A. alternata* extract (lot no. 169626) for an additional 7 h. DMSO was used as a vehicle control (VH) for caspase 3/7 inhibitor I. Fibroblast viability in the absence of *A. alternata* was also measured as a control (Nontreatment). (D) Control and IPF fibroblasts (*n* = 3, each) were stimulated with 400 *μ*g/mL *of A. alternata* extract (lot no. 276920, see Materials and Methods for the determination of *A. alternata* extract concentration) for 0.5 h, and TUNEL assay was performed. Nuclease was used as a positive control for the TUNEL response. (**) and (***) indicate *P *<* *0.01 and *P *<* *0.001 between nontreatment and nuclease treatment groups in control and IPF fibroblasts, respectively. (E) Control fibroblasts (*n* = 3) were stimulated with 500 ng/mL of FasL for 24 h, and a TUNEL assay was performed. (*) indicates a significant difference at *P *<* *0.05. Values represent the mean ± SEM as a percentage relative to nontreatment groups, set at 100%. Among 8 pairs of control and IPF fibroblasts shown in Figure 1, control fibroblasts sensitive to *A. alternata* extract*‐*dependent cell death and IPF fibroblasts that are resistant to *A. alternata* extract were selected for assays shown in Figure 2B–E.

**Figure 3:**
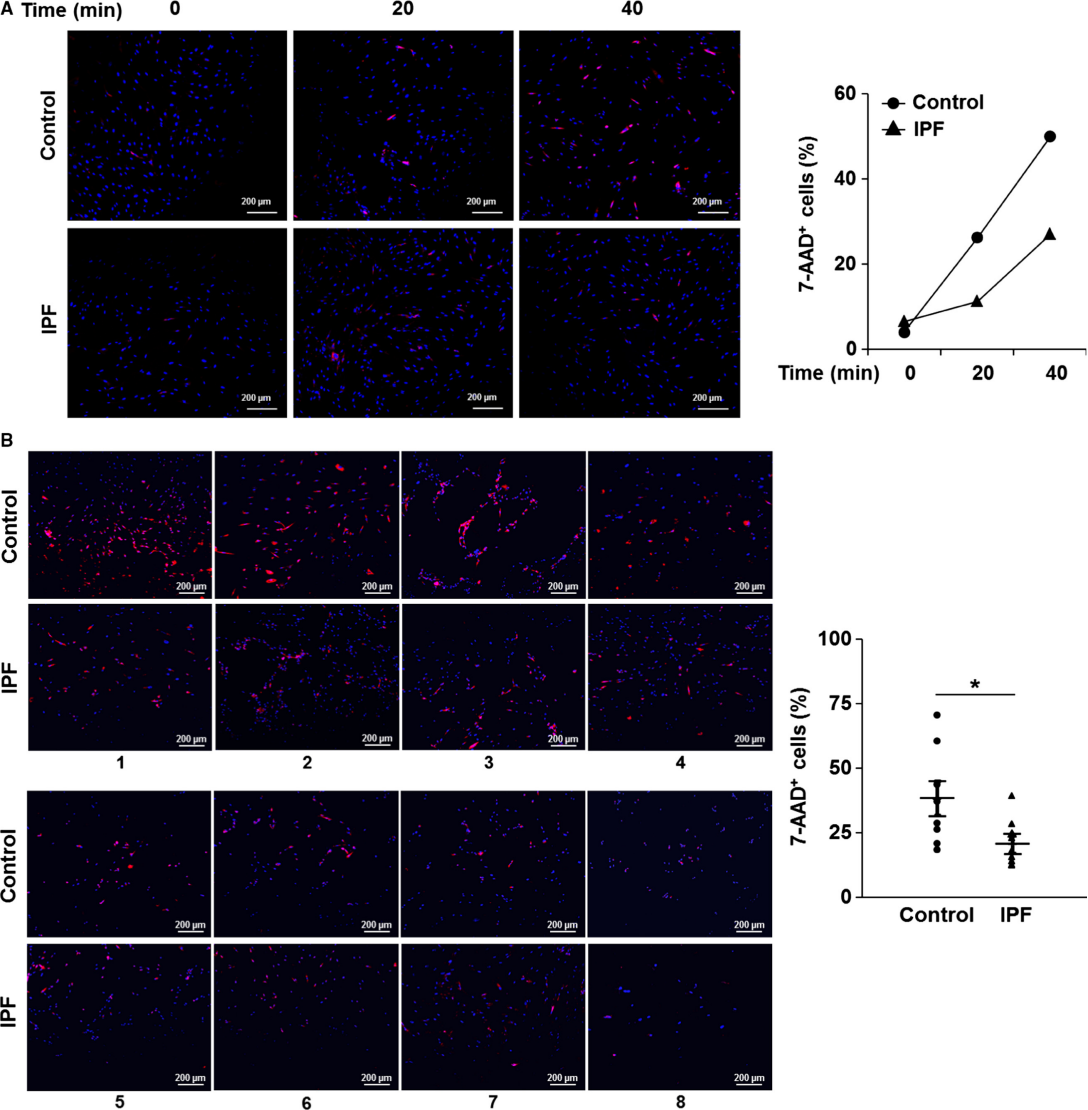
*A. alternata* extract induces necrotic cell death. (A) Control and idiopathic pulmonary fibrosis (IPF) fibroblasts were stimulated with 100 *μ*g/mL of *A. alternata* extract (lot no. 169626) for 0, 20, or 40 min at 37°C. Cells were then stained with 7‐AAD. *Left*, Fluorescence images for 7‐AAD (red) and DAPI (blue) obtained using a confocal microscope. *Right*, Values are presented as a percentage of 7‐AAD‐positive cells relative to the total number of DAPI‐positive cells at each time point. (B) *Left*, randomly selected control and IPF fibroblasts (*n* = 8, each) were stimulated with 100 *μ*g/mL of *A. alternata* extract (lot no. 169626) for 40 min, and stained with 7‐AAD together with DAPI. Fluorescence images were obtained using a confocal microscope. *Right*, each circle and triangle represents the percentage of 7‐AAD‐positive cells relative to the total DAPI‐positive cell number for each data point at 40 min after *A. alternata* extract stimulation. (*) indicates a statistical significance at *P *<* *0.05. Scale bars represent 200 *μ*m.

We next examined the underlying mechanisms of cell death‐inducing effect of *A. alternata* extract on lung fibroblasts. We first examined whether the treatment of *A. alternata* extract causes the characteristics of apoptotic cell death with enhanced caspase 3/7 activity and DNA fragmentation (Elmore 2007; Sharon et al. 2009). As shown in Figure 2A, caspase 3/7 activity was not significantly altered when control and IPF fibroblasts were stimulated with 100 *μ*g/mL of *A. alternata* extract. Our positive control assay showing that caspase 3/7 activity was increased in response to apoptosis inducing FasL in control fibroblasts under this condition supported the conclusion that cell death caused by *A. alternata* extract treatment was not due to caspase‐dependent apoptosis (Fig. 2B). To further confirm this finding, control and IPF fibroblasts were pretreated with a caspase 3/7 inhibitor I, and viability was measured in the presence of *A. alternata* extract. Caspase 3/7 inhibitor I did not affect the *A. alternata* extract–induced cell death in control and IPF fibroblasts (Fig. 2C). We next measured DNA fragmentation by TUNEL assay in control and IPF fibroblasts treated with *A. alternata* extract. Nuclease or FasL treatment used as positive controls showed significantly increased TUNEL‐positive cells (black bars in Fig. 2D and E, respectively). Unlike these findings, TUNEL‐positive cells remained unaltered when both control and IPF fibroblasts were treated with *A. alternata* extract (gray bars in Fig. 2D). These results strongly suggest that fibroblast cell death we observed in the presence of *A. alternata* extract was not caused by apoptosis. To further elucidate the cell death‐inducing mechanism, we next examined whether *A. alternata* extract promoted necrotic cell death. For this assay, we first treated control and IPF fibroblasts with *A. alternata* extract as a function of time, and 7‐AAD‐positive necrotic cells were measured. At 40 min post *A. alternata* extract treatment, enhanced 7‐AAD‐positive control fibroblasts were observed. (Fig. 3A, left and right). To further verify whether *A. alternata* extract‐dependent necrosis was suppressed in IPF fibroblasts, control and IPF fibroblasts (*n* = 8, each) were treated with *A. alternata* extract for 40 min, and necrotic cells were also measured. Relatively low 7‐AAD‐positive cells were found in the majority of IPF fibroblasts compared with that of control fibroblasts (Fig. 3B, left). Statistical analysis demonstrated that control fibroblasts showed a 1.9‐fold greater 7‐AAD‐positive cell value compared to IPF fibroblasts (38.2 vs. 20.5% in Fig. 3B, right). Collectively, these results showed that the *A. alternata* extract promoted fibroblasts cell death via necrosis, and that IPF fibroblasts are resistant to *A. alternata* extract‐induced necrotic cell death.

### Enhanced intracellular calcium levels by *A. alternata* extract have no effect on the *A. alternata* extract‐induced necrosis

**Figure 4:**
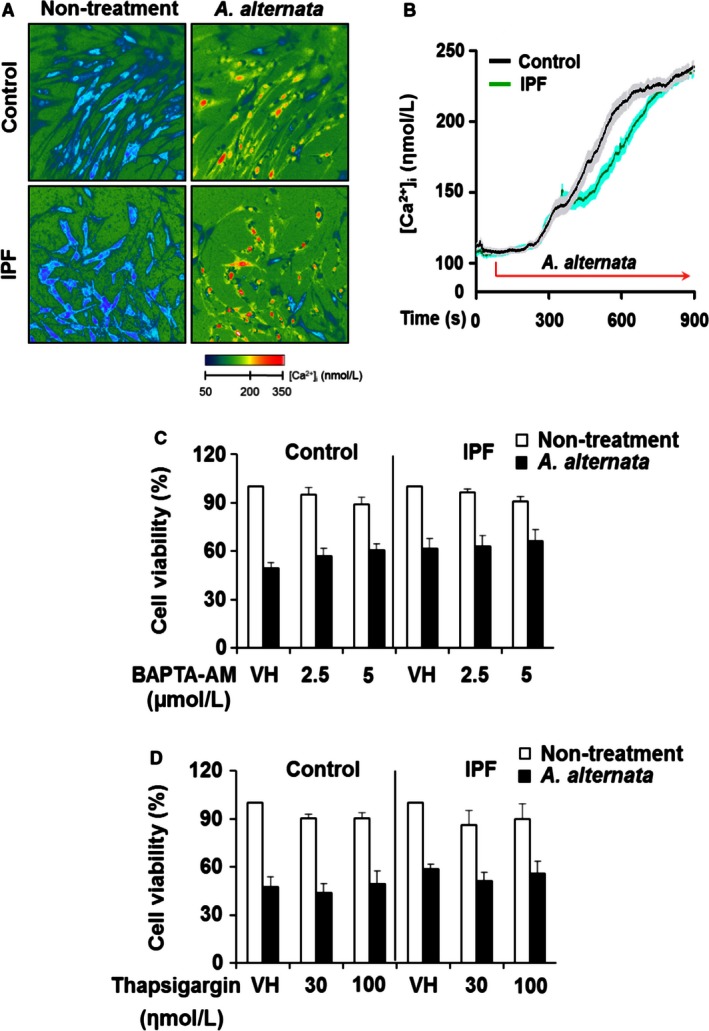
Changes in intracellular [Ca^2+^]_i_ induced by *A. alternata* extract had no effect on fibroblast cell death. Control and idiopathic pulmonary fibrosis (IPF) fibroblasts were incubated with Fura‐2‐AM for 1 h and were treated with 100 *μ*g/mL *A. alternata* extract (lot no. 169626). (A) Changes in intracellular [Ca^2+^]_i_ obtained from control and IPF fibroblasts in the presence or absence of *A. alternata* extract stimulation. (B) Corresponding time course tracings of intracellular calcium concentrations in control and IPF fibroblasts in response to *A. alternata* extract. The black line represents the mean Ca^2+^ response from a total of 60 cells from three control subjects (20 cells/each). The gray shaded region indicates the SEM for each of the mean values that constitute the solid black line. The green trace represents the mean [Ca^2+^] response of 80 fibroblasts obtained from 4 IPF patients (20 cells/each IPF patient) where the cyan shaded area indicates the SEM for the mean values that constitute the solid green line. Same control fibroblasts sensitive to *A. alternata* extract‐dependent cell death and IPF fibroblasts that are resistant to *A. alternata* shown in Figure 2B–E were chosen for these experiments. IPF fibroblast that is resistant to *A. alternata* extract shown in Figure 1 was selected for an additional IPF cell line. (C) Control and IPF fibroblasts (*n* = 3 each, 1.0 × 10^4^ cells/well) preincubated with calcium chelator, BAPTA‐AM for 0.5 h were stimulated with 400 *μ*g/mL of *A. alternata* extract (lot no. 276920) for an additional 7 h, and cell viability was measured. (D) Control and IPF fibroblasts (*n* = 3 each) preincubated with thapsigargin for 1 h were stimulated with 400 *μ*g/mL of *A. alternata* extract (lot no. 276920) for an additional 7 h, and cell viability was measured. DMSO was used as a vehicle control (VH) for BAPTA‐AM or thapsigargin. Values are the average ± SEM as the percentage against their respective non‐*A. alternata* extract treatment groups, set at 100%. Same control fibroblasts sensitive to *A. alternata* extract*‐*dependent cell death and IPF fibroblasts that are resistant to *A. alternata* extract shown in Figure 2B–E were chosen for these experiments. Fibroblast viability in the absence of *A. alternata* extract was measured under the same conditions as a control (Nontreatment).

Previous studies demonstrated that necrotic cell death can be influenced by several factors including enhanced intracellular calcium and/or ROS (Kinnally et al. 1813; Halestrap 2009; Jackson and Schoenwaelder 2010). Therefore, we next examined whether *A. alternata* extract can affect the intracellular calcium concentration. We found that *A. alternata* extract induced similar increases in [Ca^2+^]_i_ in both control and IPF fibroblasts (Fig. 4A and B). To test the role of calcium on the *A. alternata* extract‐induced necrosis, cells were treated with a calcium chelator, BAPTA‐AM or the sarcoplasmic/endoplasmic reticulum calcium ATPase (SERCA) inhibitor, thapsigargin followed by *A. alternata* extract stimulation, and cell viability was then measured. BAPTA‐AM and thapsigargin did not significantly affect the cell viability in control and IPF fibroblasts in response to *A. alternata* extract (Fig. 4C and D). These results indicate that the effect of *A. alternata* extract on Ca^2+^ mobilization in normal and IPF cells is essentially the same.

### Aberrantly low ROS generation in IPF fibroblasts is responsible for enhanced resistance to the *A. alternata* extract ‐induced cell death

**Figure 5:**
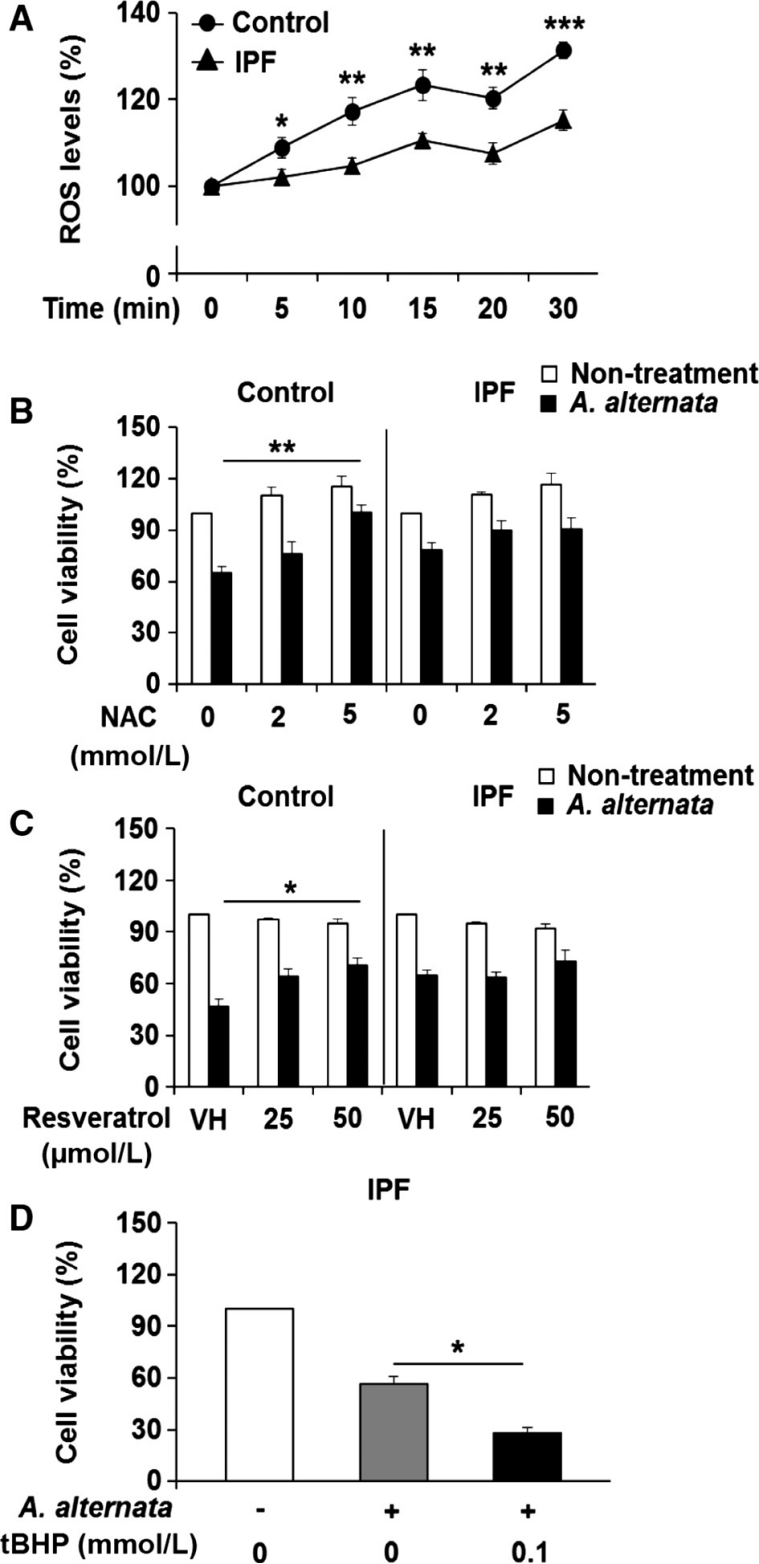
Aberrantly low reactive oxygen species (ROS) generation in idiopathic pulmonary fibrosis (IPF) fibroblasts and their resistance to *A. alternata* extract‐induced necrosis. (A) Cells were treated with 100 *μ*g/mL of *A. alternata* extract (lot no. 169626), and intracellular ROS levels were measured every 5 min up to 30 min after *A. alternata* extract stimulation. Values are the average ± SEM as the percentage against their respective nontreatment groups (0 min), set at 100%. *^,^ ** and *** showed statistical significance in ROS levels between control and IPF fibroblasts at each time point at *P *<* *0.05, *P *<* *0.01, and *P *<* *0.001, respectively. Same control and IPF fibroblasts (*n* = 8, each) shown in Figure 1 were used for this assay. (B) Control and IPF fibroblasts (*n* = 3, each) preincubated with ROS scavenger, NAC, for 2 h were stimulated with 400 *μ*g/mL of *A. alternata* extract (lot no. 276920) for an additional 7 h, and cell viability was measured. (C) Control and IPF fibroblasts (*n* = 3, each) preincubated with resveratrol for 2 h were stimulated with 400 *μ*g/mL of *A. alternata* extract (lot no. 276920) for an additional 7 h, and cell viability was then measured. DMSO was used as a vehicle control (VH). Values given in B and C are the average ± SEM as the percentage against their respective nontreatment groups, set at 100%. (*) and (**) indicate a statistical significance at *P *<* *0.05 and *P *<* *0.01, respectively. (D) IPF fibroblasts (*n* = 3, each) preincubated with 0.1 mmol/L tBHP for 0.5 h were stimulated with 400 *μ*g/mL of *A. alternata* extract (lot no. 276920) for an additional 2 h, and cell viability was measured. Values given in B–D are the average ± SEM against their respective nontreatment groups, set at 100%. (*) indicates a statistical significance at *P *<* *0.05. Same control fibroblasts showing low cell viability after *A. alternata* extract treatment and IPF fibroblasts that are resistant to *A. alternata* extract shown in Figure 2B–E were selected for Figure 5B, C and D. Fibroblast viability in the absence of *A. alternata* extract was measured under the same condition as a control (Nontreatment).

We next investigated whether alteration of intracellular ROS levels is responsible for promoting control fibroblast cell death in response to *A. alternata* extract using the DCFDA staining method. Although ROS levels were increased in both control and IPF fibroblasts in response to *A. alternata* extract stimulation*,* IPF fibroblasts showed significantly lower ROS generation compared to control fibroblasts for all time points (Fig. 5A). These results suggest that sensitization to *A. alternata* extract in control fibroblasts was due to enhanced ROS generation, and that aberrantly reduced ROS protects IPF fibroblasts from *A. alternata* extract*‐*induced cell death. To test this possibility, we first examined the effects of ROS scavengers, NAC and resveratrol on cell viability of control and IPF fibroblasts treated with *A. alternata* extract. NAC and resveratrol treatment protected control fibroblasts from *A. alternata* extract‐induced cell death while there was no significant effect on IPF fibroblast's viability (Fig. 5B and C). To examine the possibility that abnormally reduced ROS causes IPF fibroblast's resistance to *A. alternata* extract‐induced cell death, IPF fibroblasts preincubated with a ROS generation agent, *tert*‐Butyl hydroperoxide (tBHP) were treated with *A. alternata* extract, and cell viability was also measured. tbHP treatment clearly decreased viable IPF fibroblasts in response to *A. alternata* extract (Fig. 5D). Collectively, these results strongly suggest that enhanced ROS generation leads to necrotic cell death of control fibroblasts in response to *A. alternata* extract, and aberrantly low ROS generation in IPF fibroblasts is associated with resistance to *A. alternata* extract‐induced necrotic cell death.

### IPF fibroblasts maintain enhanced ATP levels in response to *A. alternata* extract, causing increased viability

**Figure 6:**
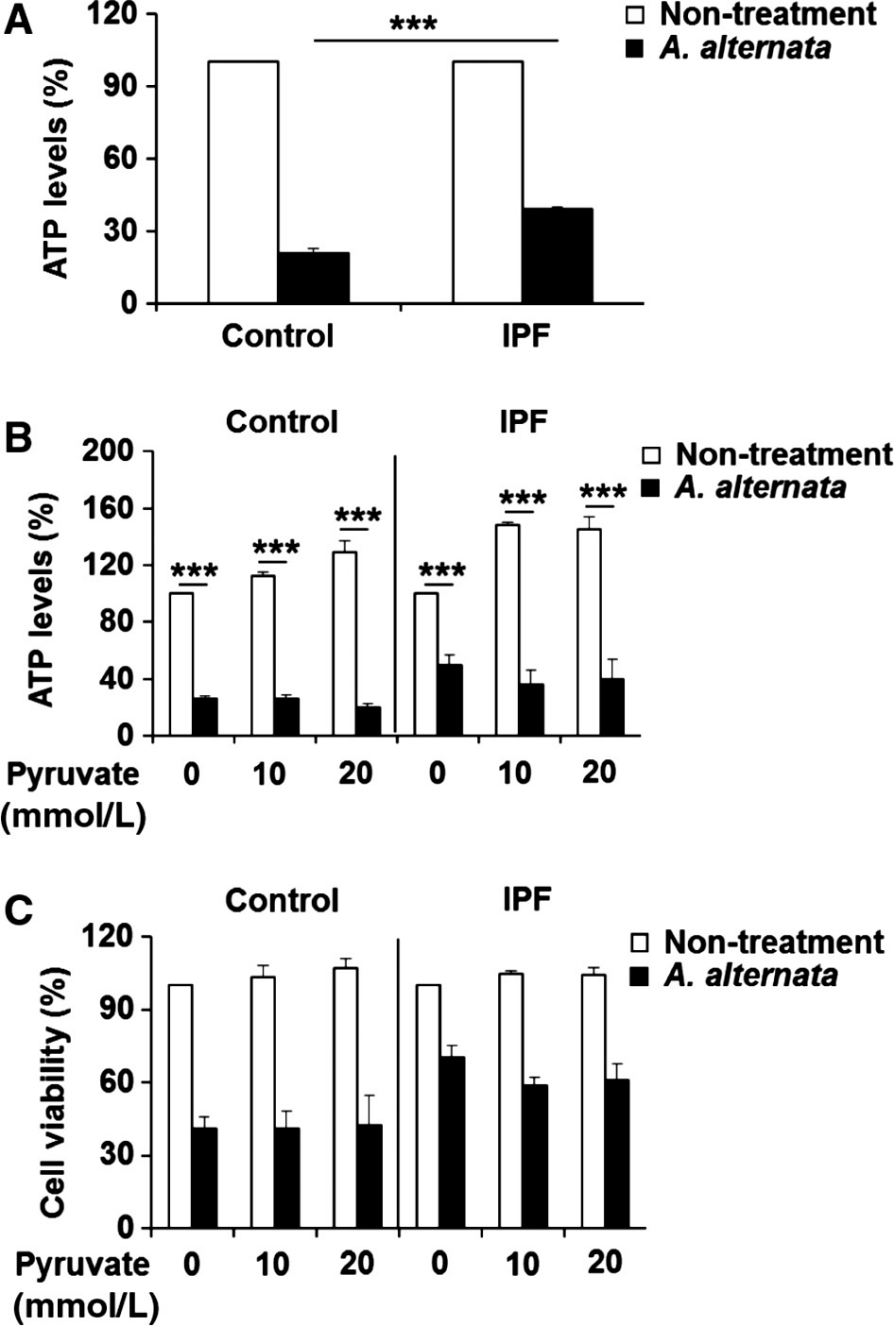
*A. alternata* extract*‐*mediated fibroblast cell death is not due to ATP‐dependent apoptosis. (A) Control and idiopathic pulmonary fibrosis (IPF) fibroblasts (*n* = 3, each) were treated with 400 *μ*g/mL of *A. alternata* extract (lot no. 276920) for 30 min. ATP levels represent the mean ± SEM as a percentage relative to their respective nontreatment groups, set at 100%. (***) indicates statistical significance at *P *<* *0.001. (B) Control and IPF fibroblasts (*n* = 3, each) preincubated with sodium pyruvate for 3 h were treated with 400 *μ*g/mL of *A. alternata* extract (lot no. 276920) for an additional 30 min, and intracellular ATP levels were measured. Values are the mean ±** SEM expressed as a percentage relative to nontreatment groups set at 100%. (***) indicates statistical significance at *P *<* *0.001. (C) Control and IPF fibroblasts (*n* = 3, each) preincubated with sodium pyruvate for 3 h were stimulated with 400 *μ*g/mL of *A. alternata* extract (lot no. 276920) for an additional 7 h. After the incubation, the cell viability was measured. Values represent the mean ± SEM as a percentage compared to their respective nontreatment groups, set at 100%. Same control fibroblasts showing low cell viability after *A. alternata* extract treatment and IPF fibroblasts that are resistant to *A. alternata* extract shown in Figure 2B–E were used for these experiments. Fibroblast viability in the absence of *A. alternata* extract was measured under the same condition as a control (Nontreatment).

It is well established that increased intracellular calcium and/or ROS accumulation disrupts the proton gradient needed for mitochondrial ATP production and subsequently results in ATP depletion, leading to necrotic cell death (Kinnally et al. 1813; Halestrap 2009; Jackson and Schoenwaelder 2010). Furthermore, apoptosis is known to be an ATP‐dependent programmed cell death process while necrosis is considered as an ATP‐independent cell death process (Eguchi et al. 1999; Sharon et al. 2009). Since our results suggest that *A. alternata* extract preferentially increases necrotic fibroblast cell death, we next measured ATP levels in these fibroblasts in response to *A. alternata* extract to confirm our findings. Although both control and IPF lung fibroblasts showed diminished ATP levels during *A. alternata* extract stimulation, IPF fibroblasts maintained approximately a twofold higher ATP level (39.2 vs. 20.9%) compared to control fibroblasts (Fig. 6A). To further clarify the role of ATP in regulating cell death, we next measured cell viability and ATP levels in response to *A. alternata* extract under conditions where ATP production was enhanced by sodium pyruvate pretreatment. Sodium pyruvate treatment alone increased intracellular ATP levels in control and IPF fibroblasts in a dose‐dependent fashion (Fig. 6B, white bars). However, sodium pyruvate pretreatment did not affect ATP levels in response to *A. alternata* extract in both control and IPF fibroblasts (black bars). Importantly, cell viability was also unaffected by various concentrations of sodium pyruvate, further suggesting that the *A. alternata* extract does not promote cell death via ATP‐dependent apoptosis (Fig. 6C). Collectively, our results consistently support the conclusion that lung fibroblasts from IPF patient are resistance to the *A. alternata* extract‐induced necrotic cell death.

## Discussion

Recent study estimated that the prevalence of fungal allergies is greater than previously believed (Martinez‐Canavate Burgos et al. 2007), and *A. alternata* infection is associated with a high risk of allergic respiratory conditions, which may lead to life threatening asthma particularly in children and young adults (Martinez‐Canavate Burgos et al. 2007). This fungal species induces immunoglobulin E (IgE)‐mediated respiratory diseases (O'Hollaren et al. 1991; Gabriel et al. 2016). Studies have shown that up to 70% of mold‐allergic patients have skin test reactivity to *Alternaria* (Bush and Prochnau 2004). Interestingly, chronic exposure to *A. alternata* has been implicated in the development of lung fibrosis (Denis et al. 2007). This study suggested that allergens such as *A. alternata* can promote lung fibrosis, and that exposure to *A. alternata* in IPF patients may worsen the disease process. Therefore, to address this possibility, we examined the potential role of *A. alternata* extract on fibroblast viability. We found that control fibroblasts exhibit enhanced cell death in response to *A. alternata* extract while lung fibroblasts derived from IPF patients are more resistant to *A. alternata* extract‐mediated cell death. Our additional assays showed that *A. alternata* mainly causes a necrosis in lung fibroblasts, and IPF fibroblasts are resistant to this asthma inducing fungus. Thus, based on these findings, a strong possibility exists that the presence of the viable fibrotic fibroblasts in patient with IPF can accelerate the fibrotic process, worsening the disease course.

Remodeling of the lung architecture is a hallmark of many lung diseases (Chetta et al. 1997). During lung remodeling, loss of alveolar walls in emphysema and subepithelial fibrosis in asthmatic airways are frequently found (Minshall et al. 1997; Bergeron et al. 2010). Although asthma is an inflammatory lung disease, studies further demonstrated that features compatible with small airways disease are common in IPF (Shaw et al. 2002; Kurashima et al. 2010). The potential link between asthma and IPF in the course of lung remodeling has previously been investigated. microRNAs, matrix metalloproteinases and cytokines such as TGF‐*β*, IL‐9, IL‐12, and PDGF, etc., associated with the lung remodeling process (Cao et al. 2000; Stone et al. 2003; van den Brule et al. 2007; Das et al. 2014; Boucherat et al. 2015) are thought to play a role in the progression of lung fibrosis. Thus, these studies suggest that the exposure to *A. alternata* initially develops asthma and may lead to the progression of lung fibrosis as disease progresses. From this perspective, it is feasible that the inhalation of *A. alternata* in patients with various stages of lung fibrosis, especially at an early stage, can increase the fibrotic process. This notion is supported by studies showing that IPF development appears to be linked to environmental factors such as allergen exposure, various chemicals, and environmental particles (Wilson and Wynn 2009). Importantly, recent preliminary investigations have shown that airborne wild‐type *A. alternata* hyphae express detectable quantities of allergen (Green et al. 2005), further suggesting that the chronic inhalation of airborne *A. alternata* can potentially promote pulmonary disease.

It is generally accepted that injury, inflammation and repair phases are a useful model to elucidate common mechanisms of pulmonary fibrosis (Wilson and Wynn 2009). In particular, it has been well described that during wound healing, the recruitment of inflammatory cells and activated (myo)fibroblasts migrate to sites of injury, facilitating the repair process (White et al. 2003; Wynn and Ramalingam 2012; Chambers and Mercer 2015). Therefore, inflammation followed by fibrosis caused by an imbalance of cell populations between *A. alternata* sensitive normal lung fibroblasts and persistent fibrotic fibroblasts may be an important pathogenic mechanism that leads to the development of lung fibrosis. Based on clinical importance of *A. alternata* on human lung health, previous attempts have been made to identify the allergenic components from *A. alternata*. Currently, there are several allergenic proteins that are characterized as allergens of *A. alternata* (http://www.allergome.org). Therefore, it may be necessary to find potential proteins that are implicated with the fibrotic process and to elucidate the precise underlying mechanisms for the prevention of the development of lung fibrosis by *A. alternata* in the future.

Previous studies demonstrated that ROS regulates both apoptotic and necrotic cell death (Kinnally et al. 1813; Sharon et al. 2009). Under the apoptosis inducing conditions, enhanced ROS activates the function of Bcl‐2 proteins such as Bak and Bax, forming pores within the outer mitochondrial membrane, causing mitochondrial outer membrane permeabilization (MOMP) (Dufey et al. 2014). The release of cytochrome C then leads to apoptosome formation, caspase 3 activation and DNA fragmentation, promoting apoptosis (Elmore 2007). In necrotic cell death, ROS induces a rapid loss of mitochondrial membrane potential by opening the mitochondrial permeability transition pore (mPTP) traversing the both outer and inner mitochondrial membrane (Kinnally et al. 1813; Jackson and Schoenwaelder 2010). This mTPT opening then breaks the proton gradient between mitochondria and cytosol needed for ATP production, finally resulting in ATP depletion and necrotic cell death (Halestrap 2009). Thus, to find the type of cell death caused by *A. alternata* extract*,* we further examined caspase 3/7 activity, DNA fragmentation, necrosis including 7‐AAD staining and ATP production in control and IPF fibroblasts. Caspase 3/7 activity was not significantly altered when control and IPF fibroblasts were stimulated with *A. alternata* extract. Moreover, when these cells were pretreated with a caspase 3/7 inhibitor, cell death was not altered in response to *A. alternata*. However, unlike these findings, enhanced necrotic cell death was found in control fibroblasts at 20–40 min after treatment of *A. alternata* extract. To confirm this finding that cell death caused by *A. alteranta* is mainly due to necrosis not apoptosis, we also performed a TUNEL assay after 30 min of *A. alternata* treatment. Our results showed that TUNEL‐positive cells remain unaltered when both control and IPF cells are treated with *A. alternata*. Taken together, our results consistently suggest that *A. alternata* extract promotes necrotic cell death in control lung fibroblasts, and the resistance of IPF fibroblasts to *A. alternata* extract‐mediated necrosis is likely due to resistance to ROS generation. Interestingly, a prior study documented that IPF fibroblasts are resistant to oxidative stress‐induced cell death (Bocchino et al. 2010), suggesting a possibility that IPF fibroblasts have acquired mechanisms that effectively protect them from oxidative stress induced cellular damage by up‐regulating antioxidant enzymes. In fact, recent findings that manganese superoxide dismutase (Mn SOD) and copper/zinc SOD are upregulated in lung tissues from IPF patients further support our concept (Lakari et al. 2000; Kinnula and Crapo 2003). Additional studies are required to verify whether these enzymes play a role in protecting IPF fibroblasts from *A. alternata*.

Although our results consistently suggest that IPF fibroblasts become resistant to *A. alternata*‐mediated cell death, there may be limitations to precisely define the pathological phenotype of IPF fibroblasts in response to *A. alternata* we observed. For example, it is inevitable that biological variability exists in the primary fibroblasts from IPF and non IPF patients due to the fact that these cells were derived from various patient groups with different biological backgrounds. To address this, we used randomly selected control and IPF fibroblasts to measure their responses to *A. alternata*, and carefully selected each fibroblast for additional assays. However, as a result of the potential existence of variable biological properties in these cells, it might be still possible that our selected fibroblasts do not completely represent the majority of control and/or IPF fibroblast phenotype in response to *A. alternata*. Perhaps, future studies using additional fibroblasts derived patient groups with similar biological backgrounds such as gender, age, smoking, etc. may address this limitation.

In summary, we showed that IPF fibroblasts are resistant to *A. alternata* extract*‐*induced cell death*,* and this resistance is due to the reduced sensitivity to *A. alternata* extract‐mediated and ATP‐independent necrosis. This alteration may be linked to the development of lung fibrosis by accelerating cell death of normal lung fibroblasts. To the best of our knowledge, this is the first study to examine the role of *A. alternata* extract on fibrotic lung fibroblast viability. Our study suggests that it may be necessary for patients with IPF to limit/reduce their exposure to *A. alternata* for the better management of lung fibrosis.

## Conflict of Interest

No conficts of interests, financial or otherwise, are declared by the authors.
